# Supplementary material for: Cocaine-induced immediate-early gene expression in the nucleus accumbens: roles of separate cAMP sensors
Source: bioRxiv. 2025 Apr 20:2025.04.15.648980. Preprint. [Version 1] doi: 10.1101/2025.04.15.648980 (PMC12190405; doi:10.1101/2025.04.15.648980)

Suppl Fig. 1 (related to Fig. 1). IEG induction after psychostimulant treatment

A: D1-MSNs signaling pathway and schematic diagram of psychostimulant treatment

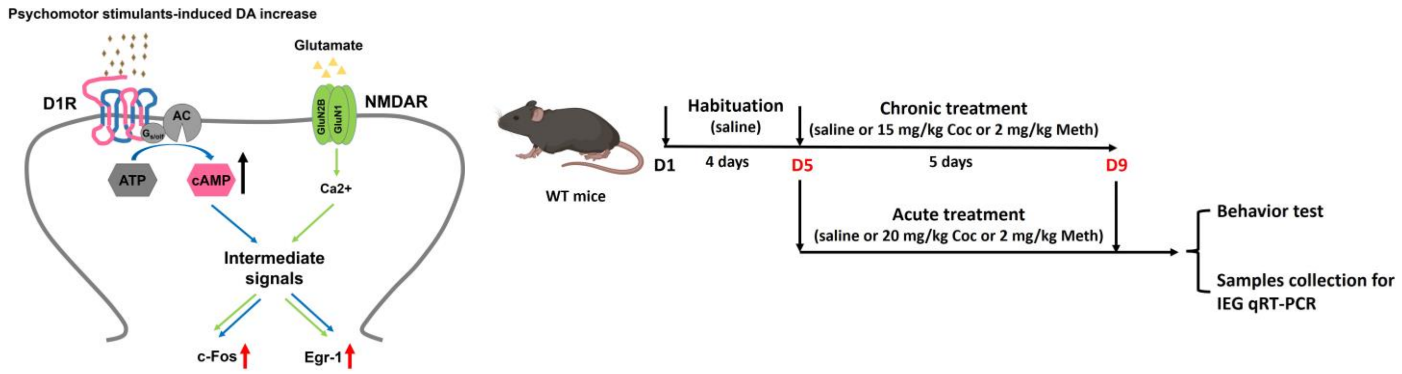

B: Cocaine and methamphetamine have similar effects on locomotor activity in WT mice

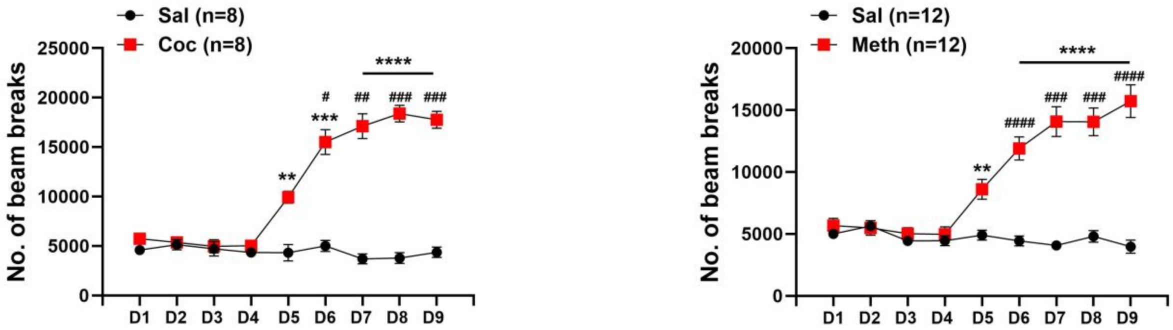

C: IEGs induction after acute and repeated psychostimulant administration measured by qRT-PCR

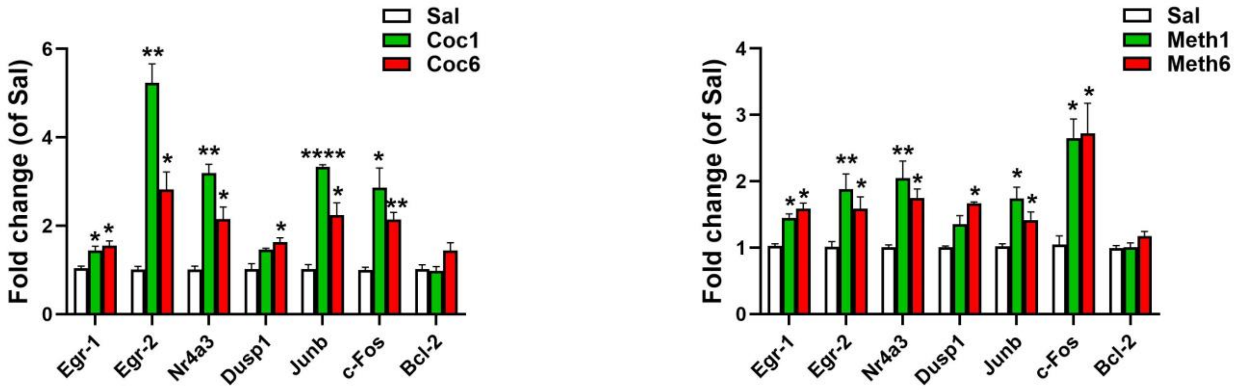

Suppl Fig. 2 (related to Fig. 1). Camk2α-Cre, but not Drd1-Cre, can penetrate LoxP locus in RapGEF2 flox mice to knock-out RapGEF2

A: RapGEF2 Exon 4 specific RT-PCR and ISH probes

RapGEF2$^{fl/fl}$ mice

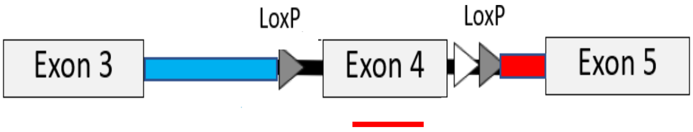

RapGEF2 FAM probe

and is also made available for use under a CC0 license.

OR BA-Mm-RapGEF2-2zz-st

B: RapGEF2 was knocked out in Camk2α-Cre^{+}::RapGEF2$^{fl/fl}$ mice, but not in Drd1-Cre^{+}::RapGEF2$^{fl/fl}$ mice

Camk2α-Cre^{+}::RapGEF2$^{fl/fl}$ mice

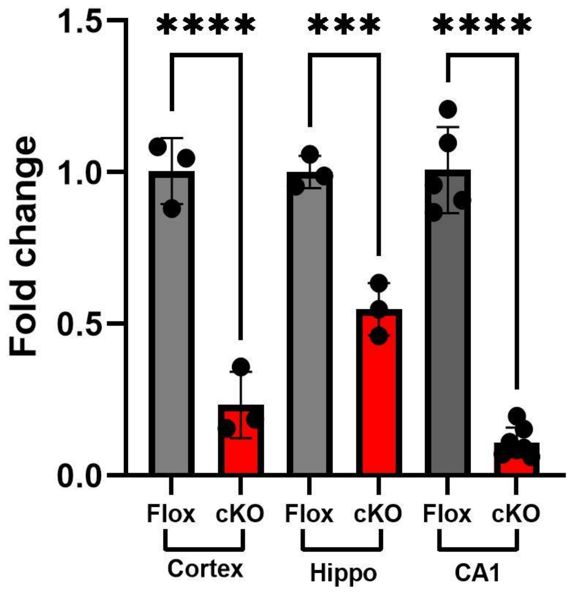

Drd1-Cre^{+}::RapGEF2$^{fl/fl}$ mice

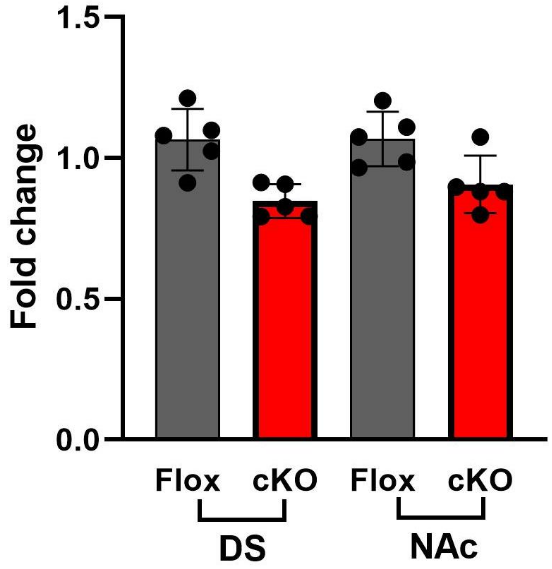

C: A decreased RapGEF2 mRNA expression was detected in hippocampus CA1 and DG, but not in CA2 and C3 in Camk2α-Cre^{+}::RapGEF2$^{fl/fl}$ mice

RapGEF2$^{fl/fl}$ (Flox) mice

Camk2α-Cre^{+}::RapGEF2$^{fl/fl}$ (cKO) mice

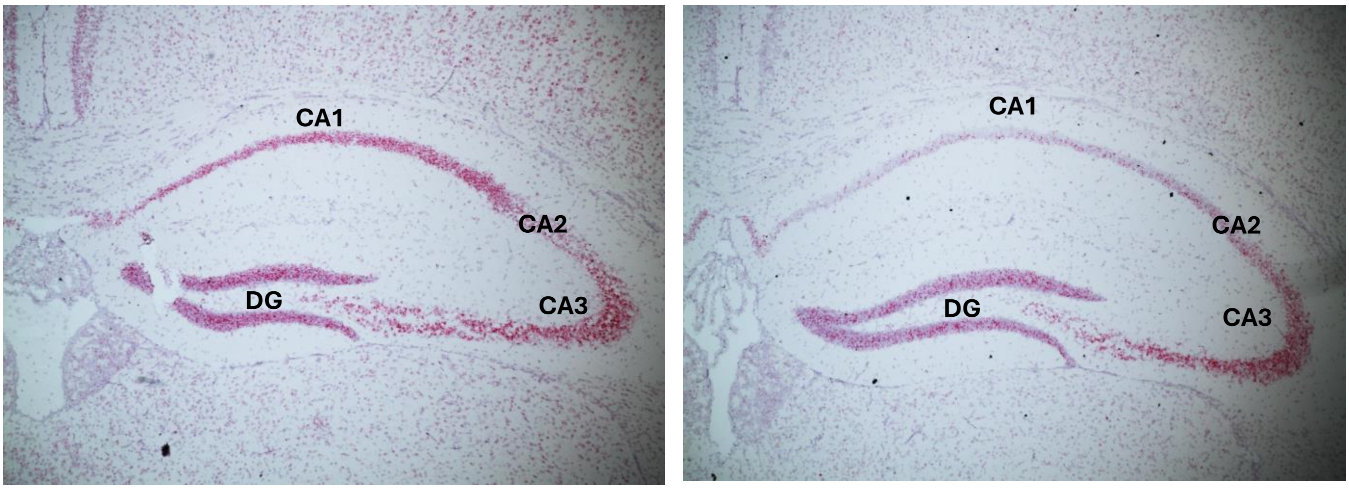

Suppl Fig.3 (related to Fig. 1): Generation of Cre amplifier mouse and D1-specific RapGEF2 knock-out mouse

A: Cre amplifier construct

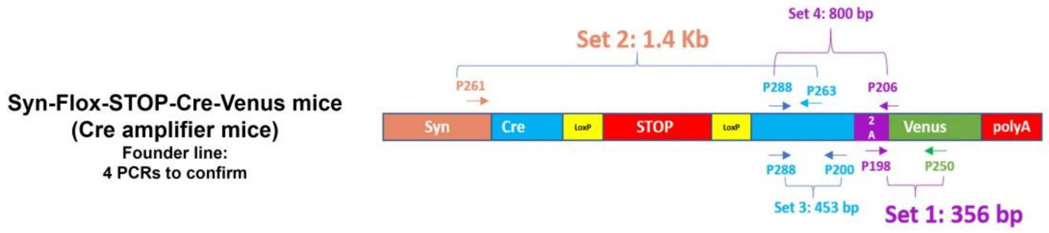

B: Generation of Drd1-Cre::Cre amplifier mouse line

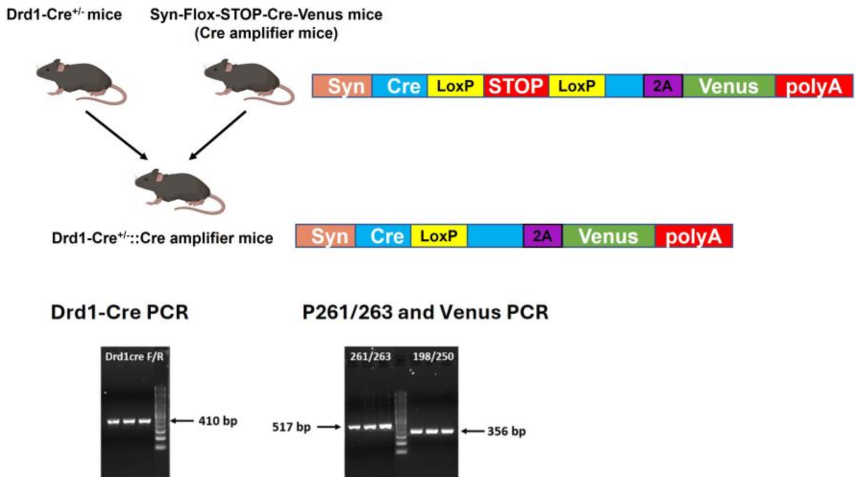

C: Venus expression in Drd1-Cre$^+$::Cre amplifier mouse

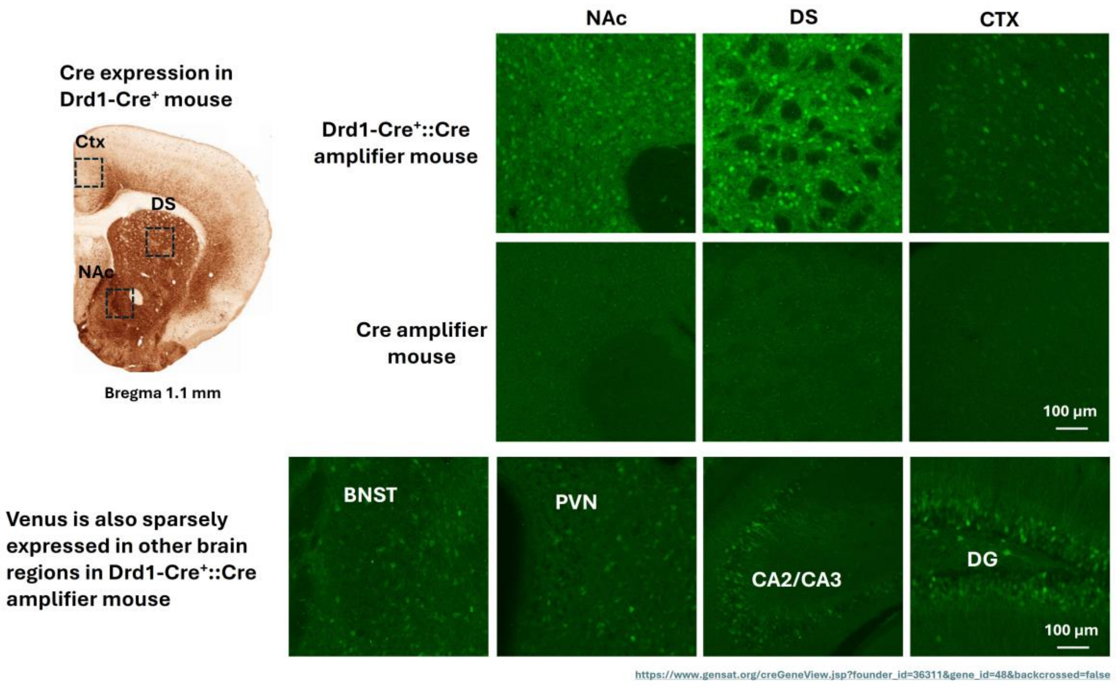

D: Generation of Drd1-Cre::CGW::RapGEF2$^{fl/fl}$ mice

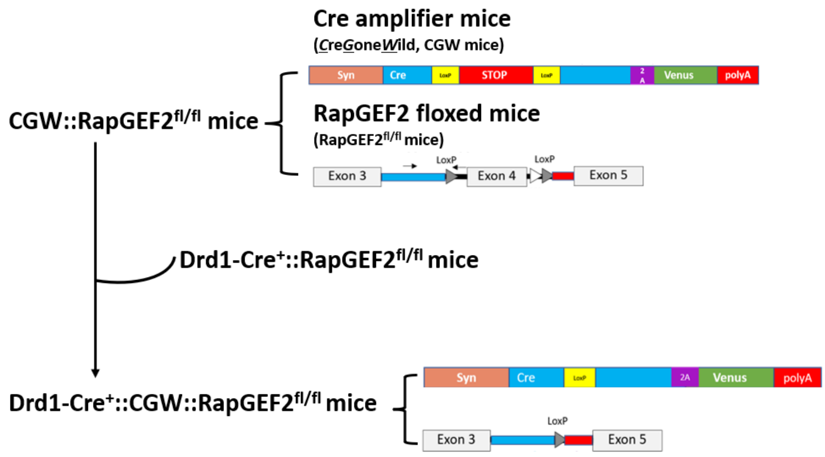

Suppl Fig.4 (related to Fig. 1): p-ERK induction pattern by various drugs in NAc

A: Representative images of p-ERK induction by various drugs from anterior to posterior NAc

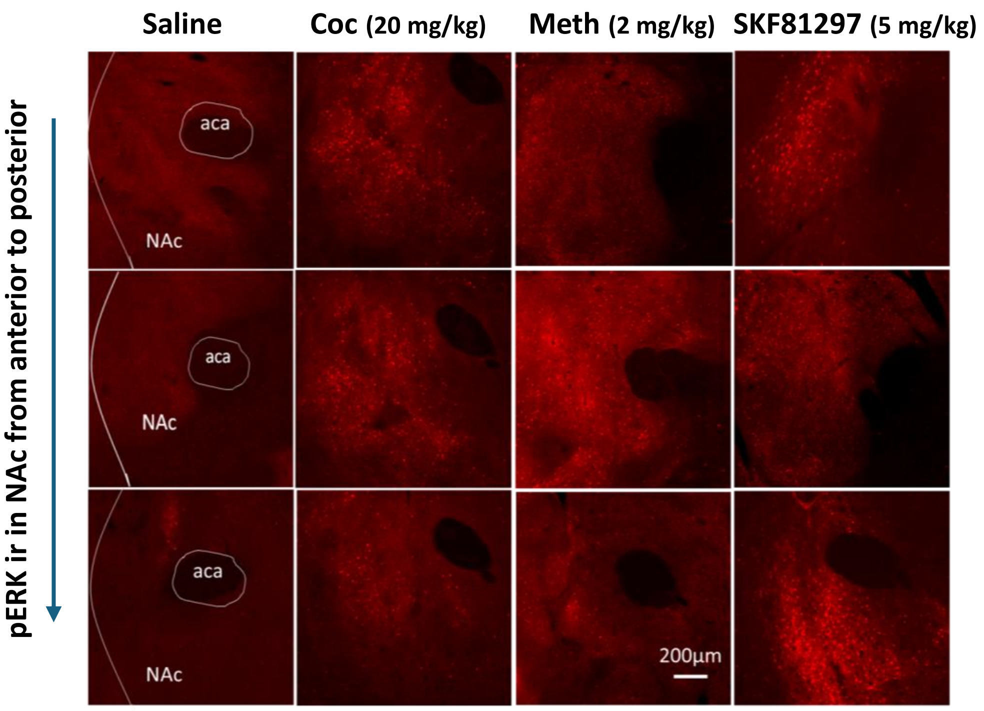

B: Topographical differences of pERK activation in serial sections of NAc by various drugs

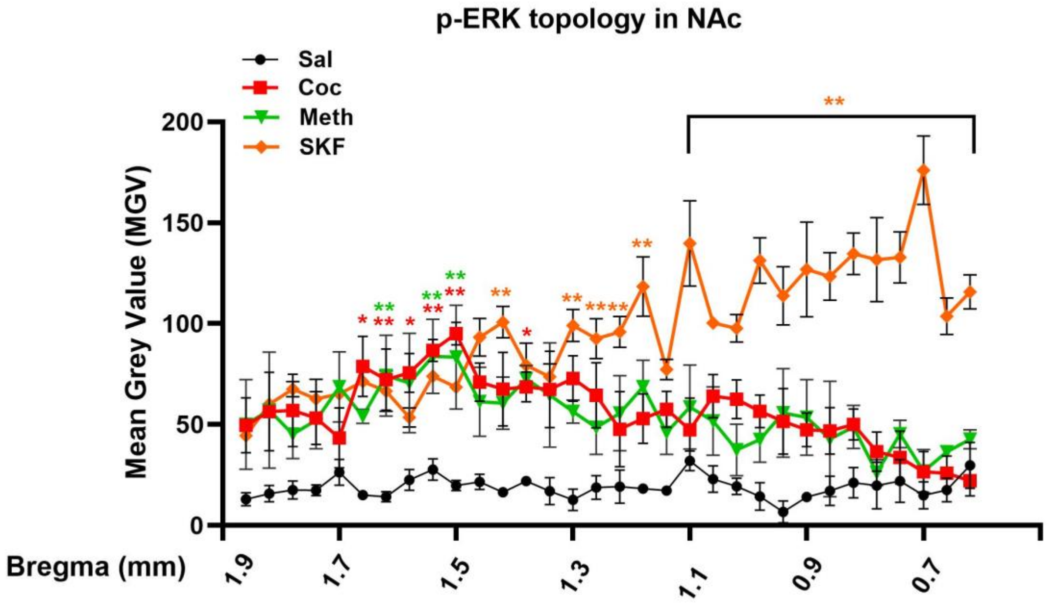

C: Regional difference of p-ERK induction by various drugs in NAc

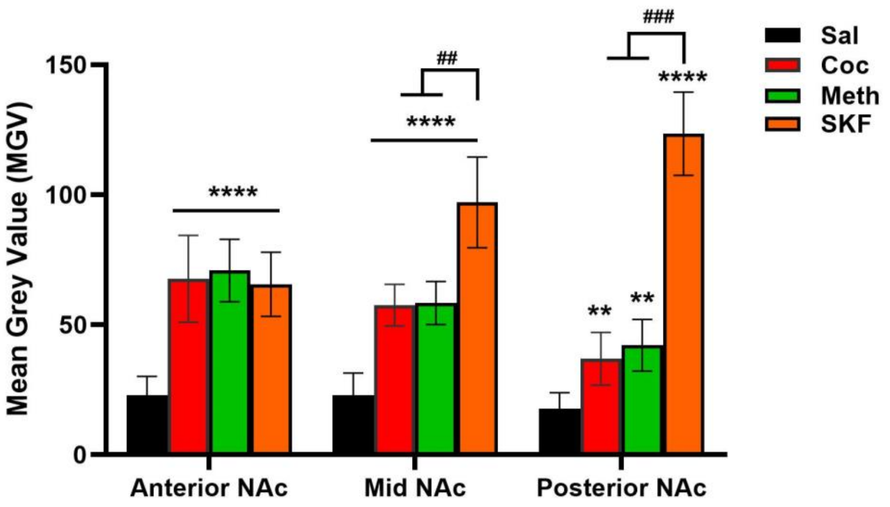

Supplement: 1 — Supplementary Figure 1. Cyclic AMP dependence of IEG induction after psychostimulant administration (A) Schematic model of D1 receptor→cyclic AMP (cAMP) metabotropic and glutamate→ calcium inotropic signaling pathway mediated immediate early genes induction in D1 medium spiny neurons (MSNs) after psychostimulant administration (Left) and acute and repeated cocaine (Coc, 20 or 15 mg/kg, i.p) or methamphetamine (Meth, 2 mg/kg, i.p) treatment for 5 days in WT mice for the measurement of locomotor activity and IEG expression by qRT-PCR (Right). (B) Mice developed robust locomotion sensitization both by cocaine (Coc, Left) and methamphetamine (Meth, Right) for 5 consecutive injections. n = 8 mice in Coc group and n = 12 mice in Meth group, **p < 0.01, ***p < 0.001, ****p < 0.0001 for Coc or Meth versus saline at each time point. #p < 0.05, ##p < 0.01, ###p < 0.001, ####p < 0.0001 for Coc or Meth D6-D9 versus D5. All panels: Mean ± S.E.D. On day 9 (D9) after 1 hr behavior recording, mice were sacrificed and NAc were dissected for qRT-PCR assay (shown in Supple Figure 1C) (C) Induction of IEG by acute (Coc1 or Meth1) or repeated cocaine or methamphetamine (Coc6 or Meth6) measured by qRT-PCR. n = 5~8 mice in each group, *p < 0.05, **p < 0.01, ****p < 0.0001 for Coc or Meth versus saline. All panels: Mean ± S.E.D. Supplementary Figure 2. Camk2a-Cre, but not Drd1-Cre, can penetrate LoxP locus in RapGEF2 flox mice to knock-out RapGEF2 (A) Design of the RapGEF2 Exon 4 specific TaqMan probe for qRT-PCR and Basescope probe for ISH, which allow us to check the RapGEF2 expression difference between control flox mice and Cre::RapGEF2fl/fl mice. (B) In Camk2α-Cre::RapGEF fl/fl mice, the relative level of RapGEF2 mRNA was ~70% decrease in the cortex, ~50% decrease in the whole hippocampus and ~85% decrease in hippocampus CA1, compared to Flox control mice. N = 5~8 mice for each group. ***p < 0.001, ***p < 0.0001 for cKO versus Flox mice. All panels: Mean ± S.E.D. Similarly, detection [file NIHPP2025.04.15.648980v1-supplement-1.pdf]
